# A lower-back focused motion capture and electromyography dataset of Australian sheep shearers at work

**DOI:** 10.1038/s41597-025-05102-9

**Published:** 2025-05-23

**Authors:** Mark Robinson, Tomislav Baček, Ying Tan, Denny Oetomo, Chris Manzie

**Affiliations:** 1 The University of Melbourne, Department of Mechanical Engineering, Melbourne, 3010 Australia; 2 The University of Melbourne, Department of Electrical and Electronic Engineering, Melbourne, 3010 Australia

**Keywords:** Databases, Musculoskeletal system, Biomedical engineering

## Abstract

Lower back pain, the predominant cause of years lived with disability, is widespread among various occupations characterised by prolonged and repetitive movements, including healthcare, agriculture, and construction. Despite this prevalence, evidence on the time-dependent changes in muscular function and corresponding motor control systems that contribute to pain remains limited, largely due to the lack of experimental data collected in real-world environments over extended periods. This paper addresses this gap by introducing an open dataset from an intensely physical and repetitive activity—sheep shearing—collected from eleven shearers in Australia. Data on participants’ movement, muscle activity, and self-reported fatigue were recorded in their natural work environment throughout an entire day, divided into four 2-hour sessions. The dataset encompasses a wide range of experiences, including shearers with prior back injuries and those using back support harnesses. This unique dataset enables the exploration of time-dependent factors in pain-inducing repetitive movements, providing insights applicable to similar occupational activities where task demands can lead to chronic pain and injuries.

## Background & Summary

Lower back pain is the leading cause of disability worldwide (data up to 2020^1,2^), with more than 80% of people expected to experience back pain in their lifetime^3^. Lower back disorders are especially prevalent among occupations that require postures of prolonged or repetitive spinal and hip flexion with legs mostly remaining straight, known as *stooped work*^4^. These include, among other, construction, mining, agriculture (e.g., sheep shearing), and healthcare (nursing).

Effective management of lower back pain requires a complex approach that involves multiple aspects and factors. This is evidenced by the leading model for understanding and treating lower back pain—the biopsychosocial (BPS) framework^5–7^, which includes considerations from three separate yet interconnected aspects: biomechanical, psychological, and social. Among these, biomechanical factors can easily be measured (e.g., using off-the-shelf sensors) and used as quantitative biomarkers for injury^7^. This makes biomechanical factors an attractive—and potentially the best—candidate for identifying indicators capable of predicting lower back injury^8^. Notably, a clear progression of biomechanical forces to tissue pathology and pain^9^ can greatly assist in understanding the cause and the development of injury.

The biomechanical approach to understanding and managing lower back pain is predicated on the knowledge of the working musculature and associated neuromuscular control. For example, the muscles surrounding the lumbar spine prevent the head, cervical, and thoracic spine from collapsing^10^, making these muscles crucial in virtually every scenario involving upper body movement. Achieving this requires sophisticated neuromuscular system that delivers both stiff and flexible movements across six degrees of freedom (DoFs) irrespective of the task. A healthy neuromuscular system readily delivers these movements through rapid modulation of the relevant muscle forces. When either the musculature or its neuromuscular control become compromised, continued activity causes the body to compensate, thereby increasing the likelihood of injury.

Research suggests that exposing musculature and the associated neuromuscular system to repetitive and prolonged sub-acute stress, rather than to a single large exposure, is the predominant cause of lower back injuries^11–13^. In sheep shearing in particular, injury risk increases throughout the day, with 68% more injuries occurring in the last two hours of work compared to the first two hours of work^14^. As the task progresses over the course of day, fatigue starts kicking in, leading to altered kinematics^15^ and changes in neuromuscular control^16^. These time-dependent changes in kinematics (i.e., biomechanics) and neuromuscular control of relevant musculature play crucial roles in the etiology of these injuries^17^, with motor control deficiencies being primary predictors for the onset of such disorders^18^. However, experimental studies validating these points over longer periods of time—necessary to induce fatigue and related phenomena as discussed above—are largely lacking, underscoring a significant gap in our understanding of how these deficiencies correlate with back pain and injury^8^.

Advancements in technology now enable long-term monitoring studies of muscles in real-world applications, such as stooped work. These studies allow for the analysis of musculature loading patterns and changes in corresponding neuromuscular control, tracing the evolution of lower back injuries. By exploring task-specific muscle and motor control changes using biomechanics, data collection over extended periods becomes crucial for advancing our comprehension of how sustained stress influences lower back pain development. The challenge lies in conducting long-term studies in real workplaces, as it is difficult to maintain controlled data collection without imposing on workers’ ability to effectively carry out their job. Consequently, there is a pressing need for long-term real-world datasets that can help us understand the links between motor control deficiencies, ongoing stress, and chronic back pain, potentially guiding the creation of more tailored preventative and therapeutic strategies.

This work provides such a dataset of biomechanical data collected from eleven human subjects over an entire 8-hour working day of stooped working in a real workplace setting. Specifically, this dataset captures activity of sheep shearing, a widespread and quintessential stooped work in Australia. With 3000 active shearers as of 2019^19^ and characterised by an incidence rate of six-times the industrial average in Australia^20^, sheep shearers suffer from high risk of lower back pain and injuries. Additionally, a shearer who works for 10 years has a more than 50% chance of sustaining a permanent injury due to their working conditions^21^, making them an ideal population for study.

By documenting the physical demands of sheep shearing over a full day and under actual working conditions, this dataset:

- allows for a detailed and elaborate **observations of time-dependent factors within motor control data** in a stooped activity, and
- serves as a **vital resource for understanding stooped work** activities beyond sheep shearing, used here as an archetypical case.

The findings from these data can aid in the improvement or development of quantitative biomarkers, providing valuable insights into mitigating the high incidence of work-related injuries in physically-demanding jobs.

## Methods

### Participants

Eleven (11) male sheep shearers (age 32 ± 13 years, height 1.83 ± 0.08 meters, mean  ± standard deviation) were recruited from the Australian Wool Innovation (AWI) network of shearers for this study. Per subject information can be found in Table 1, along with additional information that was considered relevant to this dataset. Specifically, this includes the history of back injury and use of a ceiling-supported back-harness, an assistive device that reduces the load on the lower back^22,23^.

**Table 1:** Subject Information.

Subject	Age	Height	Experience	Previous back injury	Harness use	Shed design
1	24	1.92 m	3 years	No	No	Catching-pen
2	54	1.71 m	36 years	Yes	Yes	Catching-pen
3	33	1.76 m	8 years	No	No	Catching-pen
4	24	1.86 m	4 months	No	No	Catching-pen
5	21	1.67 m	2 weeks	No	Yes	Catching-pen
6	23	1.89 m	11 months	No	No	Catching-pen
7	61	1.86 m	40 years	Yes	Yes	Catching-pen
8	35	1.90 m	3 years	No	Yes	Catching-pen
9	27	1.91 m	5 years	No	Yes	Catching-pen
10	25	1.85 m	6 years	No	No	Raised race
11	30	1.81 m	12 years	No	No	Raised race

AWI advertised the study within their shearing contractor network and passed on details of shearing locations and times when shearers showed interest in participating. Responsible researchers then attended the participating shearing sheds and confirmed with shearers in person whether they would agree to be monitored while shearing. Written consent was obtained from all shearers who agreed to participate. As shearers are paid per sheep sheared on the day, they were compensated A$100 for potential lost time during the day. There were no specific exclusion criteria; if the shearers were healthy and skilled enough to shear professionally, they were deemed eligible for the study. By agreeing to participate in the study, shearers agreed to their data being used in scientific publications and related future research. The experiment was approved by the University of Melbourne Human Ethics advisory group (Ethics ID 1853436). Two of the shearers (Subjects 2 and 7) had suffered previous back injuries/pain, and both sheared with the assistance of a harness. Subject 2 does not suffer pain while shearing but reported experiencing pain while standing up straight for extended periods. Subject 7 would start experiencing significant pain after only an hour or two of shearing without using a harness but was mostly pain-free while using a harness.

### Experimental study

The dataset was collected via an observational study of sheep shearers performing their usual tasks while at work. A brief explanation of sheep shearing is included, followed by the protocol description.

#### Sheep shearing

**Fig. 1:**
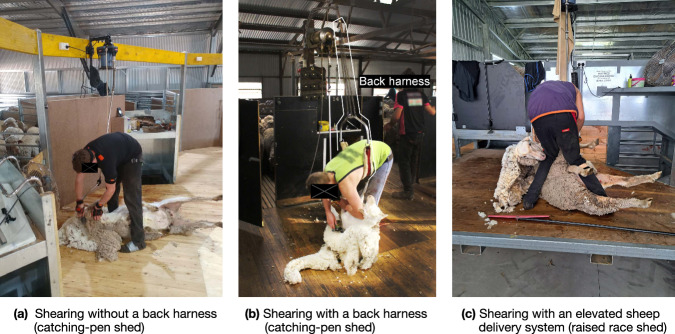
Typical sheep shearing environment. Sheep are held in a pen (**a,b**) or a race (**c**) on the side of the shearing stand (workspace) and are dragged out by the shearer into the shearing position (depicted here). The infrastructure and mechanical plant for powering the shearing tool are provided on-site. Shearers choose whether or not they want to use a back harness. Figure on the left shows a shearer with the motion capture system – Xsens inertial measurement units (orange boxes). Muscle activation sensors, also worn by the shearers, are not visible in this figure.

Sheep shearing is a repetitive stooped work occupation, with sheep shearers as an itinerant workforce that shears on-location at woolgrowers’ properties. The woolgrowers provide the infrastructure and typically the necessary mechanical plant for powering the shearing tool. Shearing is comprised of two main sub-tasks. The first is the catch and drag, where a shearer is required to gain control of a sheep and drag it backwards to their shearing stand. In a (traditional) catching-pen shed (Fig. 1a,b), this involves entering a catching-pen (a closed space that holds the sheep) to then catch the sheep, flip it, and drag it back to the stand, while in a raised-race shed (Fig. 1c), shearers grab the sheep from a narrow, elevated space (race) adjacent to the shearing stand and drag it a short distance to the stand. In theory, the more recent raised-race design should make the catch and drag easier, by greatly reducing the drag length and allowing gravity to assist with flipping the sheep. However, this is still a significant manual handling task, with a typical sheep weighing around 70 kilograms^24^. The second and main sub task is the actual shearing, where the shearer must control and maneuver the sheep into different positions that are amenable to using the mechanical hand-piece to separate the wool from the animal. The shearing task requires the shearer to be in a stooped position (bent over at the waist with straight or nearly straight legs) and control the sheep with their legs and off-hand while dragging the hand-piece over the sheep in a series of shearing *blows*^25^. The majority of the time is spent in the shearing task, resulting in the shearer maintaining a stooped posture for around six (out of eight working) hours each day^26^. Figure 1 shows a typical shearing environment and stooped position: catching-pen shed with (Fig. 1b) and without (Fig. 1a) the harness, and a raised race shed (Fig. 1c). Harness is a spring-loaded structure that hangs above the shearing stand and loops around the shearer’s torso providing a small level of support. A typical shearer in Australia will shear from 140-180 sheep per day, with highly skilled (*gun*) shearers managing upwards of 200–250 per day, but this is dependent on the type of sheep being shorn. There are also elite shearers who can shear more than this, with the world record for merino ewes at 540 in eight hours^27^.

**Fig. 2:**
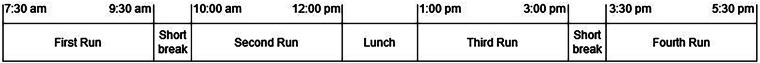
Shearing rest-work periods.

The standard shearing day in Australia starts at 7:30 am, and shearing takes place in four two-hour sessions (i.e., *shearing runs*). There is typically a one hour lunch break between runs 2 and 3, and a 30-minute break between runs 1 and 2, and runs 3 and 4. This is illustrated in Fig. 2. IMPORTANT: Due to the interruptions, five out of eleven (5/11) shearers only did the first three of the four two-hour shearing runs (Subjects 2, 5, 6, 10, 11). Additionally, one shearer (Subject 1) was recorded over three consecutive days, and these data are included as two extra subject-days. With this, the dataset consists of 13 shearer-days of data collected from 11 shearers with 5 shearer-days consisting of 3 shearing runs.

#### Protocol

As an observational study, the protocol had to minimally affect the typical shearing work-rest periods outlined above. For this reason, all equipment placement and sensor calibrations had to be done prior to the first 2-hour shearing run. To comply with this, shearers were asked to be at their shearing stand 45 minutes early at the start of the day, at 6:45 am. Equipment used included Inertial Measurement Unit (IMU) based motion capture system (Xsens Awinda, Movella, Netherlands) and surface electromyography (sEMG) Trigno Avanti sensors by Delsys, USA. Hardware synchronisation between the two systems was performed with the Delsys trigger module. The sEMG sensors had a 10 mm inter-electrode distance and were sampled at at 2148 Hz. The IMU motion capture output was sampled at 60 Hz.

As per Table 1, some shearers use a ceiling-mounted back harness as part of their equipment to reduce or eliminate the pain while shearing, as shown in Fig. 1b. It is the shearer’s choice to use a harness and it was considered unethical to require them to forgo the use of the harness during data collection. Five shearers elected to use a back harness, and a note was made of this for the later analysis.

**Fig. 3:**
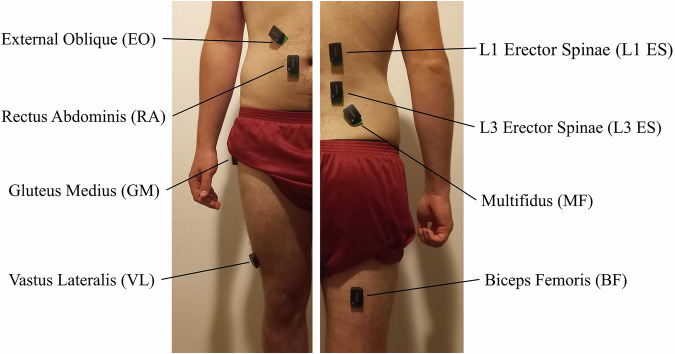
Locations of surface electromyography (sEMG) sensors. Each shearer was instrumented with 16 sEMG sensors in total, eight per side. To keep them from falling, sensors were secured with the kinesiology tape (not shown).

Upon arriving to their work stations, shearers were instrumented with 16 sEMG sensors placed bilaterally on eight muscles: the Erector Spinae at the level of the first and third lumbar vertebrae (L1 & L3 ES), Multifidus at the level of the fifth lumbar vertebrae (L5 MF), Rectus Abdominis (RA), External Oblique (EO), Gluteus Medius (GM), Vastus Lateralis (VL), and Biceps Femoris (BF) (Fig. 3). Due to the study taking place in shearing sheds, sensors were placed with subjects standing naturally as opposed to the *subject posture during placement* guidelines. The L1 ES, L5 MF, GM, VL, and BF sensors were placed bilaterally in accordance with the SENIAM guidelines^28^. The ES muscle sensors were placed laterally to the L3 spinous process (L3 ES), the RA muscle sensors 3 cm lateral to the navel^29^, and the EO muscle sensors 15 cm lateral to the navel, just below the rib cage^30^. The skin was prepared by rubbing an alcohol solution on the sensor locations. To hold all sensors in place for the full work day, each sensor was additionally secured with the kinesiology tape.

**Fig. 4:**
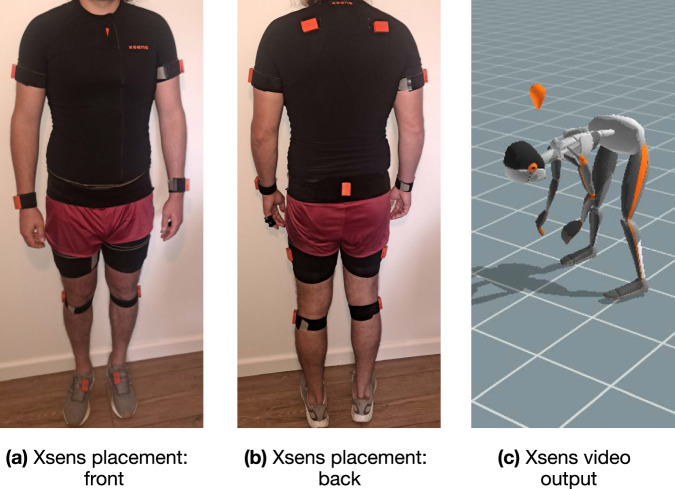
Xsens motion capture system. **(a)** Xsens sensors, as seen from the front of the subject. To showcase the shearing sensor placement, the tape is missing and the sensors are moved to the outside of the straps. **(b)** Xsens sensors, as seen from the back of the subject. To showcase the shearing sensor placement, the tape is missing and the sensors are moved to the outside of the straps. **(c)** The video output of the motion capture system. A scaled avatar of the subject performing the recorded motion from on-body sensors.

After placing the sEMG sensors, the subjects’ body-segment lengths were measured according to the Xsens guidelines. Measurements included ankle, knee, hip, shoulder, and full-body height. Furthermore, foot length, arm-span, and hip and shoulder width were also measured and used to scale the model in the Xsens software. Once body segments were measured, 17 IMUs were placed using Velcro straps, tight-fitting shirt, and head-band, all provided by Xsens. The head-band, shirt, and some of the straps can be seen in Fig. 1a—straps can be seen around the wrist and upper-arm, with tight fitting shirt worn on the torso; orange sensors can also be seen. The lower body sensors are covered by the shearers regular work-wear, with foot-located sensors taped to specialty shearing shoes, with example sensor placements demonstrated in Fig. 4a, 4b. All sEMG sensors are covered by clothes. The lower body sensors were placed under the shearers’ clothes and additionally secured with the tape, to prevent movement while in contact with the sheep. The IMUs were placed according to the Xsens guidelines (see Table 2), with the exception of the lower leg sensors. The recommended placement on the shin-bone was not suitable for sheep shearers, as the inside of both legs are required to hold and manoeuvre sheep during the work. This would expose the IMUs to large external forces and cause significant discomfort for the shearer. Thus, these IMUs were re-positioned to the outside of the leg. The Xsens guidelines state that all sensors can be placed anywhere on each body segment, as localisation is part of the calibration process. However, the recommended sensor locations are designed to minimise skin and sensor movement relative to the skeleton, so a small reduction in accuracy is expected relative to laboratory conditions. Good calibrations were still achieved as per the software, and visual inspection of the motion capture avatar.

**Table 2:** Xsens guidelines on IMU sensor placement.

Sensor	Explanation
Head	Place the sensor inside the headband and place it around your head. Place the sensor with the long edge in the direction of the band for stability of the sensor. The orientation of the headband on the head is not important as long as the sensor is stable.
Shoulder	Place the sensors in the Velcro patches in the back of the t-shirt. For better attachment of the sensor to the shirt place it horizontally so the entirety of the sensor contacts the Velcro patch.
Upper arm	It is recommended to place the sensor on the lateral side of the upper arm in between the muscle groups of the biceps and the triceps. In this way, the movement of the sensor due to movement of the muscles can be reduced.
Forearm	Place the sensor closer to the wrist, as this provides more information about pronation and supination rotations than if the sensor was placed higher on the forearm. Also, right above the wrist area has less fatty tissue, decreasing the chances of skin motion artifacts.
Pevis	For pelvis motion measurement, tighten the pelvis strap (largest strap provided) around the pelvis bone at the height of the anterior superior iliac spine. Place the sensor on the first layer of the strap and underneath the second layer if possible, and vertically oriented. Please make sure the sensor is placed on the pelvis, not high on the waist.
Upper leg	Place on the middle of the lateral thigh, as this has less probability of having fatty tissue and also reduces the chance of the strap sliding down. Be careful to not leave the tip of the Velcro in the inner part of the leg, since when moving it can easily scratch with the other leg and loosen.
Lower leg	On the tibia, close to the knee.
Foot	Place the sensor on the foot pad and place it inside your shoe. The sensor should be placed on the front upper part of the foot, in between the ankle joint and the toes joint. The sensor should be placed on the front upper part of the foot, in between the ankle joint and the toes joint. The sensor can also be place with tape. The only requirement is that it will stay fixed in place. (For the data collection these were taped onto the shearers’ specialty footwear as the laces are not like normal).
	Note: There is generally no specific orientation needed for the sensors in the Awinda system.

After the sensors were placed, the sEMG calibration was performed. The sEMG sensors were calibrated using a series of maximum voluntary contractions (MVCs)^28,29^. For the L1 ES, L3 ES, and L5 MF sensors, a standing isometric back extension exercise was performed with the torso flexed at 45^∘^, manually braced by a researcher. Despite not being a typical method for producing a MVC, this approach was used for practical reasons. Namely, due to the dirty floor in the shearing shed environment from dust and ovine urine, a prone position could not be used. Consequently, this might result in contractions less than the typical MVC. However, submaximal voluntary contractions (SVCs) have been shown to be appropriate for normalising sEMG signals in back muscles^31,32^. In particular, Dankaerts *et al*.^32^ demonstrated that maximal and sub-maximal voluntary contractions both showed excellent within-day reliability in healthy and patients (ICC range 0.75-0.98), with sub-MVC showing better within-day performance, thus providing evidence that sub-MVC is suitable for normalisation of trunk muscle excitation signals. The order of muscles in MVC tests is as subsequently described. For the RA and EO muscles, a sit-up position was adopted with a 45^∘^ torso angle and manual bracing provided by a researcher, as in^29^. The subject was instructed to flex maximally as if completing the sit-up, then attempt to twist right and left. For the GM muscle, the subject was instructed to lie on each side and abduct their hip against the provided bracing^29^. For the VL and BF muscles, the subject was instructed to sit in a chair or on the raised shearing boards, grasp the edge of the surface, and sequentially flex and extend each knee against the bracing provided at the ankle^28^. Manual bracing was deemed sufficient from a consistency perspective as the researcher was able to ensure no movement from the subject, effectively delivering an isometric maximal contraction. The sEMG signal was normalised to the highest amplitude for each muscle achieved in any of the exercises. Each MVC was conducted three times, with a 5 second hold and at least 10 seconds rest between each repetition.

The motion capture system was then calibrated using the procedure recommended by Xsens. This involves the subject standing in a static pose before naturally walking a short distance forward and returning to the original location and position. The calibration is achieved in the software by minimising errors over the dynamic movement. In addition to the main calibration of the system in the early morning, before the start of the first shearing run, motion capture was also re-calibrated before the start of other three daily runs (2–4). To do so, shearers were required at their stand 15 minutes before the run, where the same procedure was repeated as in the morning (static pose standing and a short walk). The motion capture IMUs were recharged during the lunch break (between runs 2 and 3), which required taking them off and putting back on, but this was compensated for by the re-calibration, as mentioned above.

Finally, at the start and end of each run, shearers were asked to rate their perceived level of fatigue using a modified Borg CR10 scale^33^, seen in Table 3. The shearers were given an explanation of the table at the start of the day and were shown Table 3 on paper each time they were required to answer. The results can be seen in Table 4.

**Table 3:** Modified Borg CR10 Scale for self-reported muscle fatigue.

Score	Explanation
1	Very weak
2	Weak - Fatigue is barely noticeable
3	Some fatigue
4	Some fatigue - Muscle fatigue is notable, it would be nice to slow down a bit
5	Strong fatigue - Tired and hard. It would be nice to take a rest, but you could shear another run
6	Strong fatigue - Tired and hard. You would like a rest, but you still do not have difficulties going on
7	Very strong fatigue - The muscle fatigue is so strong that you wish to stop and rest
8	Very strong fatigue - You have a strong desire to rest
9	Very strong fatigue - The muscle fatigue is nearly the worst you’ve experienced
10	Extremely strong fatigue - The muscle fatigue is the worst you have ever experienced before

**Table 4:** Self-reported fatigue data for the start and end of all shearing runs using the scale in Table 3.

Shearer-day	Start R1	End R1	Start R2	End R2	Start R3	End R3	Start R4	End R4
S1 Tues	2	2	3	3	6	7	7	8
S1 Wed	3	3	3	4	7	7	8	8
S1 Thurs	3	3	5	5	7	8	9	9
S2 Thurs	2	6	6	8	5	8	N/A	N/A
S3 Tues	2	4	2	4	2	4	3	6
S4 Wed	2	3	3	4	3	6	5	7
S5 Thurs	1	2	1	5	3	7	N/A	N/A
S6 Fri	2	3	3	5	2	6	N/A	N/A
S7 Mon	1	1	1	2	1	2	2	3
S8 Tues	3	5	5	6	6	6	6	8
S9 Wed	1	2	2	4	3	4	4	7
S10 Thurs	2	2	2	2	2	4	N/A	N/A
S11 Fri	1	1	1	1	1	2	N/A	N/A

### Data outputs

Kinematic data were collected using the Xsens Awinda portable motion capture system. The system uses a fusion of the accelerometer, gyroscope, and magnetometer channels in each sensor, which, combined with a scaled skeletal model, outputs joint angles at 60 Hz in the Xsens MVN Analyse software. Using IMU sensors is practical in shearing sheds due to uncontrollable external factors and the presence of natural light, which would interfere with marker-based system relying on reflective marker surfaces (e.g., Vicon). The IMU-based motion capture full-body system provides a visual output based on a biomechanical model, as shown in Fig. 4c. Data from the IMUs are collated in the Xsens MVN Analyse software and used as inputs to a kinematic biomechanical model and fused using an Extended Kalman Filter. This is done with several assumptions on the behaviour of ground-contact points depending on the settings in the software. The result of the ground-contact points assumptions is the system that may not register movement in the intertial frame if the ground contact point(s) slide(s) across the floor. This would happen if, e.g., feet are dragged or the person is sitting in a sliding chair. Consequently, some drift was noted in the subject centre of gravity (CoG) location in *X* and *Y* axes in the intertial frame. Importantly, this does not cause errors in the joint angles in a body-centred frame. From the model, joint angles are supplied in three (3) degrees of freedom for each joint. Segment shapes for the avatar are standardised but scaled based on body-segment measurements taken from the participants at the start of each day.

The sEMG sensors provide raw data at 2148 Hz. The raw sEMG signals were filtered with a 2^*nd*^ order Butterworth filter with a pass-band between 20–450 Hz. Then, the sEMG envelope (ENV) was calculated by rectifying the filtered sEMG signal and low-pass filtering with a cutoff frequency of 6 Hz^34^. This envelope signal was normalised to the maximum value of the envelope from the MVCs collected for that day’s shearing. This produced the filtered signal and the EMG envelope from each sEMG sensor, in addition to the raw EMG. The kinematic data are extracted from the MVN Analyse software at 60 Hz, which outputs joint angles, and raw data including: segment orientations, positions, velocities, and accelerations, segment angular velocities, and accelerations, foot contact points, as well as sensor raw accelerations, magnetic fields, and orientations.

## Data Records

**Fig. 5:**
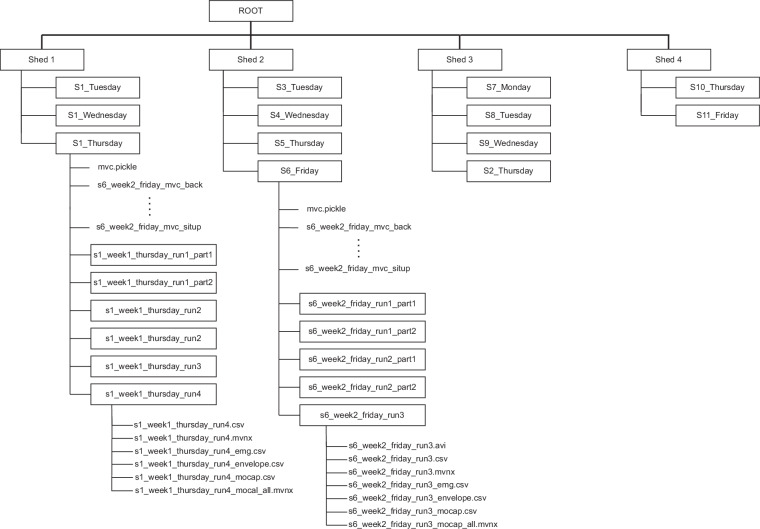
Overview of the data hierarchy. At the highest level, data are organised into sheds, with each shed corresponding to a different branch (location). Within each shed except Shed 1, data are separated into days corresponding to different shearers (in Shed 1, all three days come from the same shearer). For each individual day, data includes MVC files in the root folder and, in most cases, one folder per shearing run. Where a run had to be interrupted, it is separated into multiple files, e.g., *run1_part1* and *run1_part2*. Within each folder/run, six files can be found, including .csv and .mvnx formats. The data stored in each can be found in Table 5. Some folders/runs come with a video file *_runX.avi* containing video output (animated avatar) from the motion-capture software. This is present for subjects that were used in training or evaluating the human-activity-recognition algorithm developed in^25^ that was used for the data segmentation.

The data described in this work are stored on figshare^35^. The structure of the data within the repository can be seen in Fig. 5. Within the root folder, the data are separated into four sub-folders, each corresponding to a different data collection location (shearing shed). Within the shed sub-folders, individual subject’s data are organised into separate folders. With the exception of Subject 1, whose data were collected on three separate days, each Subject sub-folder also corresponds to a different shearing day. Within each sub-folder corresponding to a shearer (shearing day), there are multiple .CSV files containing the MVC EMG data collected right after placing the sEMG sensors. The files correspond to the five (5) MVC movements, as indicated by their name (e.g., *s6_week2_friday_mvc_back.csv*). In addition to these files, each Subject (shearing day) folder contains sub-folders pertaining to the shearing runs, generally one folder per run. In some cases, where data recording was interrupted (e.g., a sensor came off), the runs are split into multiple parts, resulting in multiple sub-folders labelled with an additional *_partX* in the folder name. Where interruptions did occur, there is at least a few minutes gap in the data in most cases as interrupting the shearer was not safe and re-calibration of the motion-capture system was needed.

Data files, summarised in Table 5, can be found in the *_runX* (or *_runX_partX*) sub-folder. The entirety of the movement and muscle activation data are stored in files with *_emg.csv*, *_envelope.csv*, and *_mocap.csv* ending. For completeness, the files containing the outputs of the Delsys and Xsens software are also included.

**Table 5:** Explanation of the low-level files.

Filename	Contents
_runX(_partX).avi*	Video file containing an animated avatar output from the motion capture software
_runX(_partX).csv	Raw EMG output from Delsys software (header-row at 20th row)
_runX(_partX).mvnx	XML tree motion capture output: joint angle-based only
_runX(_partX)_emg.csv	Filtered EMG in tabular format (header-row at first row)
_runX(_partX)_envelope.csv	Normalised EMG envelope in tabular format (header-row at first row)
_runX(_partX)_mocap.csv	Mocap output in tabular format (header-row at first row)
_runX(_partX)_mocap_all.mvnx	XML tree motion capture output: all outputs including raw IMU data

*The .avi file is only present for some subjects and in some runs.

All three (3) files containing muscle activity data (raw, filtered, and calculated envelope) and one file containing the motion capture data (*_mocap.csv*) come with two labels, corresponding to two separate shearing sub-tasks. These subtasks make up one task iteration repeated over the working period and include catch-and-drag and stooped shearing, as mentioned in the Sheep Shearing sub-section. The data are segmented into these sub-tasks on an event-driven basis using a Hidden Markov Model-based classifier^25^ developed from partially labelled data from six subjects. For each of these subjects, one morning and one afternoon run were used in training and testing, carried out firstly with a 75:25% train:test split, and also through the leave-one-person-out (LOPO) method for training and evaluation. The sub-task labels (*catch and drag* and *shearing*) allow dataset users to develop and test other supervised human-activity-recognition (HAR) methods in a real workplace environment using the provided IMU and/or sEMG data. It is worth noting that data are mostly auto-labelled, albeit there are cases where manual labelling had to be applied. In the case of the former, labels are stored under the column headed *HMM_labels*. In the case of the latter, labels are stored under the column headed *Labels*, with unlabelled data (rows) taking a value of *NaN*.

### Data issues

Several issues are present in the collected data. The centre of gravity output from the Xsens motion capture system is known to slowly drift over time as a result of the assumptions used in the model. This does not affect joint-angles on the body-centred frames, but the CoG in the inertial frame will drift around 15 m over a two-hour run.

It was noted earlier in this section that some of the shearing runs come in multiple parts within the collected data. This was due to the interruption of the motion capture, including a physical impact dislodging an IMU or a shearer habitually adjusting some clothing, which would move a sensor out of calibration. When interruptions occurred, the motion capture system required re-calibration, leading to gaps in the data in the middle of runs. To re-calibrate the system, the shearer needed to complete the current sheep and then redo the calibration procedure for the motion capture system. Depending on the shearer’s skill and how close they were to finishing the current sheep, this would take anywhere from 2 to 10 minutes.

Several issues in the raw data were also identified in an analysis and summarised in Table 6. Consistently, the motion capture body’s centre of gravity (CoG) drifted in the X and Y directions in the inertial frame. This is a known issue due to the assumptions present in the whole-body model, as previously discussed. This drift occurs slowly over the two-hour run and could be partially mitigated by *resetting* the CoG location at the end of each shearing phase. Namely, the shearer releases the finished sheep down a chute in the same location where they pick one up each time. Additionally, the motion capture had some issues with the ball of foot angles in various shearers. The IMU sensors are susceptible to external forces from the sheep causing high accelerations, which can show up as movement after the data fusion takes place. This was observed with high angles (outliers) showing up in the ball of foot angles for some shearers, as the feet are often in contact with the sheep to hold it in place for shearing. Similar outliers were observed for some shearers’ right shoulders. Impacts on that shoulder don’t appear to be commonplace, but this is the arm that holds the shearing hand-piece.

**Table 6:** Issues in the raw motion capture and electromyography data.

Sub-Day	Data	Run 1	Run 2	Run 3	Run 4
**S1** Tue	**EMG**	—	—	—	VL Left clipping at end of run
	**MoCap**	X,Y CoG drift; outliers in ball of foot angles	X,Y CoG drift; outliers in ball of foot angles	X,Y CoG drift; outliers in ball of foot angles	X,Y CoG drift; outliers in ball of foot angles
**S1** Wed	**EMG**	EO Right clipping	None	EO Right clipping	EO Right clipping
	**MoCap**	X,Y CoG drift	X,Y CoG drift	X,Y CoG drift	X,Y CoG drift
**S1** Thu	**EMG**	EO Right clipping	EO Right clipping	VL Left clipping	—
	**MoCap**	X,Y CoG drift; outliers in ball of foot angles; outliers right shoulder angles	X,Y CoG drift; outliers in ball of foot angles; outliers right shoulder angles	X,Y CoG drift; outliers in ball of foot angles; outliers right shoulder angles	X,Y CoG drift; outliers in ball of foot angles; outliers right shoulder angles
**S2** Thu	**EMG**	—	—	EO left silent; L1, L3 Left minor clipping	N/A
	**MoCap**	X,Y CoG drift; outliers in ball of foot angles	X,Y CoG drift; outliers in ball of foot angles	X,Y CoG drift; outliers in ball of foot angles	N/A
**S3** Tue	**EMG**	EO Right some clipping; VL Right some clipping	—	EO Left silent; EO Right some clipping; RA Left, Right some clipping	EO Left silent; RA right some clipping
	**MoCap**	X,Y CoG drift; outliers in ball of foot angles; outliers right shoulder angles	X,Y CoG drift; outliers in ball of foot angles; outliers right shoulder angles	X,Y CoG drift; outliers right shoulder angles	X,Y CoG drift; outliers in ball of foot angles; outliers right shoulder angles
**S4** Wed	**EMG**	RA Left clipping; RA Right clipping	RA Right clipping; L1 ES Left some clipping	GM Left clipping; RA Right clipping	RA Right some clipping
	**MoCap**	X,Y CoG drift; outliers in ball of foot angles	X,Y CoG drift	X,Y CoG drift; outliers in ball of foot angles	X,Y CoG drift; outliers in ball of foot angles
**S5** Thu	**EMG**	RA Right clipping; L3 ES Left clipping	EO Left and Right removed for discomfort; L3 ES some clipping	EO Left and Right removed for discomfort; RA Right and Left clipped	N/A
	**MoCap**	X,Y CoG drift	X,Y CoG drift	X,Y CoG drift	N/A
**S6** Fri	**EMG**	—	—	—	N/A
	**MoCap**	X,Y CoG drift; outliers in ball of foot angles	X,Y CoG drift	X,Y CoG drift; outliers in ball of foot angles	N/A
**S7** Mon	**EMG**	VL Right clipping	VL Right silent	VL Right silent	VL Right silent
	**MoCap**	X,Y CoG drift	X,Y CoG drift	X,Y CoG drift	X,Y CoG drift
**S8** Tue	**EMG**	—	—	L3 ES minor clipping	—
	**MoCap**	X,Y CoG drift	X,Y CoG drift; outliers in ball of foot angles	X,Y CoG drift; outliers in ball of foot angles	X,Y CoG drift
**S9** Wed	**EMG**	—	—	—	—
	**MoCap**	X,Y CoG drift	X,Y CoG drift	X,Y CoG drift	X,Y CoG drift
**S10** Thu	**EMG**	GM & EO silent	GM & EO silent	GM & EO silent	N/A
	**MoCap**	X,Y CoG drift	X,Y CoG drift	X,Y CoG drift	N/A
**S11** Fri	**EMG**	GM & EO silent	GM & EO silent	GM & EO silent	N/A
	**MoCap**	X,Y CoG drift	X,Y CoG drift	X,Y CoG drift	N/A

There are also some issues noticed with the raw EMG recordings. For some shearers and some muscles, the EMG signal would occasionally go silent or experience clipping (measured activity is higher than the sensor output so the signal saturates – it is *clipped*). This is not uncommon in wireless sensors, which will inevitably drop some packages, especially during long sessions of data collection like in this case. Furthermore, the nature of sheep shearing prevents researchers from intervening when a sensor experiences a minor issue, potentially impacting signal quality around the time of the issue. Two shearers asked for the EO sensors to be removed due to them feeling discomfort wearing sensors and safety harness at the same time. While present, these issues were rarely noticed in the lower back muscles, as these generally experienced lower level muscle activity. Importantly, these sorts of issues do not have large effects on the per-sheep analyses, like the ones shown in Fig. 8, but may show up as additional noise.

## Technical Validation

**Fig. 6:**
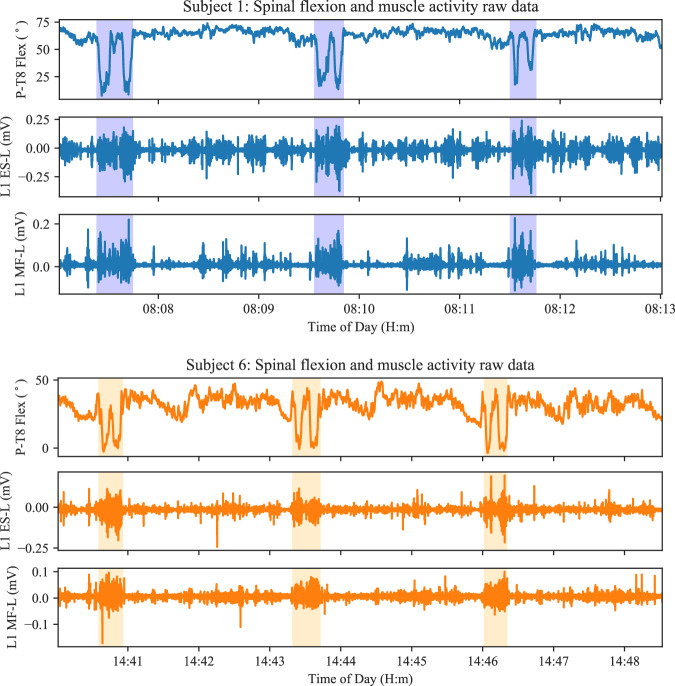
Lumbar flexion and raw EMG from back muscles on the left-hand-side are plotted for Subject 1 & 6. The catch-and-drag portion of shearing is highlighted in each plot. Higher muscle activity can be seen in the catch-and-drag task and periods of flexion-relaxation are seen during the stooped (non-highlighted) shearing task. Flexion-relaxation is known to occur in most people while stooping, and has been noted previously in sheep shearing. EMG bursts are still observed where disturbances are present or torso movement is needed while stooping.

While sEMG and motion capture data has been collected in sheep shearing previously^36,37^, the datasets are not available publicly and the time-period of data collection is short (less than 15 minutes per case). Hence, there is no direct comparison that can be made to this data. In the absence of a direct comparison, we present a number of extracts of data in order to assess validity using motor control principles known in the literature. The flexion-relaxation phenomenon is present during stooping^17,38^ and has been previously noted in sheep shearing^36,39^. It is explained that during the shearing task, shearers use a stooped posture and small torso movements occur during this phase^36^. This is demonstrated in Fig. 6, where spinal flexion and muscle activity (raw EMG) are plotted for two subjects over a time-period where each subject shears three sheep. The highlighted sections of the plots correspond to the catch-and-drag portion of the task, and the remaining sections to the stooped shearing sub-task. The flexion-relaxation phenomenon is present in the stooped posture, but muscle activity is noted periodically during the stooped shearing task. This muscle activity is likely related to the small movements around the stooped *base* posture. There is a much lower level of lower back muscle activity observed during the shearing task when compared to the largely upright catch-and-drag task, indicating the flexion-relaxation phenomenon. The increase in flexion during the middle of the catch-and-drag task that can be seen in Fig. 6 is indicative of the shearer bending down to catch the sheep in the pen.

**Fig. 7:**
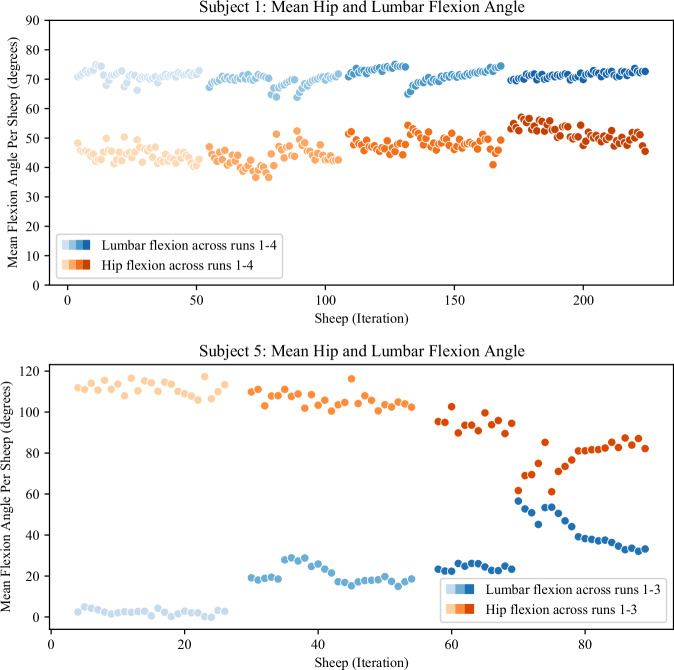
Mean hip and lumbar flexion angle per sheep for Subject 1 & 5. Each datum is the mean value of flexion from the shearing task of that sheep. The two subjects have different skill levels and shear at different rates, resulting in different numbers of sheep (per run, and in total). Subject 5 (bottom figure) performed only 3 runs as shearing was interrupted on that day. Forward flexion in stooped work comes from the combination of hip and lumbar flexion as the legs remain mostly straight, and different contributions from the hips and back can be seen with different subjects. This is part of the lumbo-pelvic rhythm, which is known to differ between people, and change under fatigue. It can be seen to change over the course of the day, in a different way for each subject shown here.

**Fig. 8:**
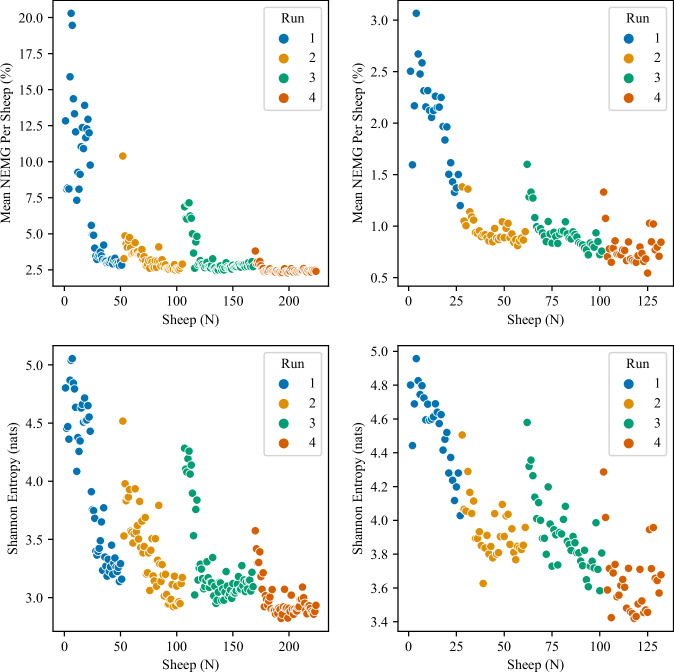
Mean nEMG and Shannon Entropy per sheep in the L3 Erector Spinae muscle on the right-hand-side (L3 ES-R) is plotted for Subject 1 & 6. The mean nEMG feature is the mean EMG across the shearing task of each sheep normalised to each subject’s MVC. The Shannon entropy feature is calculated from the filtered EMG signal segment taken from the shearing task of each sheep. The time-dependency in these motor-control features can be clearly observed, with the values falling across each run, and then also across the day. Periods where the feature ‘recovers’ are also noted after the short- and long-breaks that are present in the shearing day.

Figures 7 and 8 show features extracted from the data after segmentation using the HAR model^25^. In Fig. 7, the hip and lumbar flexion can be seen from two subjects. The relative contributions of hip and lumbar flexion form part of a person’s lumbo-pelvic rhythm^40^, which is known to vary between people and under fatigue. Differing contributions of the two movements in each shearer can be seen, whereby the relative contributions from the hip and back vary in opposite directions and in differing amounts across the two shearers.

In Fig. 8, two features extracted from the L3 ES-R muscle are presented for two shearers. Both the L3 ES-R Mean Activation and L3 ES-R Shannon Entropy features reduce across the four shearing runs, with recovery in both features noted following the breaks between shearing runs. Previous analysis of this data indicates that the decrease across runs is statistically significant, with *p* < 0.01 across the sampled population^41^. Example data from two subjects with these two features is shown here to demonstrate the time-dependence within the sEMG data, which is expected given the literature around motor-control and indicates the usefullness of longer-term collection of these data.

## Data Availability

The activity labels in the data-set (where not manually labelled) have been generated using an HMM-based HAR model^25^. Custom code has also been used to generate the EMG envelope. This code is provided with this paper and can be found on GitHub website.
